# Towards a Simplified Estimation of Muscle Activation Pattern from MRI and EMG Using Electrical Network and Graph Theory

**DOI:** 10.3390/s20030724

**Published:** 2020-01-28

**Authors:** Enrico Piovanelli, Davide Piovesan, Shouhei Shirafuji, Becky Su, Natsue Yoshimura, Yousuke Ogata, Jun Ota

**Affiliations:** 1 Department of Precision Engineering, The University of Tokyo, Hongo, Tokyo 113-8656, Japan; su@race.t.u-tokyo.ac.jp; 2 Department of Biomedical, Industrial and Systems Engineering, Gannon University, Erie, PA 16541, USA; piovesan001@gannon.edu; 3 Research Into Artifacts Center for Engineering (RACE), School of Engineering, The University of Tokyo, Tokyo 113-8656, Japan; shirafuji@race.t.u-tokyo.ac.jp (S.S.); ota@race.t.u-tokyo.ac.jp (J.O.); 4 Institute of Innovative Research, Tokyo Institute of Technology, Midori-ku, Yokohama 226-8503, Japan; yoshimura@pi.titech.ac.jp (N.Y.); ogata@cns.pi.titech.ac.jp (Y.O.); 5 Integrative Brain Imaging Center, National Center of Neurology and Psychiatry (NCNP), Kodaira, Tokyo 187-0031, Japan; 6 Precursory Research for Embryonic Science and Technology (PRESTO), Japan Science and Technology Agency (JST), Kawaguchi, Saitama 332-0012, Japan

**Keywords:** MRI, EMG, graph theory, electrical network, muscle activity, forearm

## Abstract

Muscle functional MRI (mfMRI) is an imaging technique that assess muscles’ activity, exploiting a shift in the T2-relaxation time between resting and active state on muscles. It is accompanied by the use of electromyography (EMG) to have a better understanding of the muscle electrophysiology; however, a technique merging MRI and EMG information has not been defined yet. In this paper, we present an anatomical and quantitative evaluation of a method our group recently introduced to quantify its validity in terms of muscle pattern estimation for four subjects during four isometric tasks. Muscle activation pattern are estimated using a resistive network to model the morphology in the MRI. An inverse problem is solved from sEMG data to assess muscle activation. The results have been validated with a comparison with physiological information and with the fitting on the electrodes space. On average, over 90% of the input sEMG information was able to be explained with the estimated muscle patterns. There is a match with anatomical information, even if a strong subjectivity is observed among subjects. With this paper we want to proof the method’s validity showing its potential in diagnostic and rehabilitation fields.

## 1. Introduction

In the study of neuromuscular degenerative disease and in the development of rehabilitation therapies to treat them, monitoring the activity of muscles is crucial to better understand the nature of the impairment and to have a feedback about the changes occurring after applied treatments. As a consequence of pathological conditions, it is also not so uncommon to observe changes occurring in the physical structure and in the behaviour of muscles of impaired individuals [1,2,3]. It is, therefore, crucial to have a full vision that simultaneously encloses the underlying morphology and the muscle activation pattern, in order to have a full understanding of the impairment status. This overall vision would allow clinicians to have a wider picture of the pathological status, potentially being able to provide a targeted treatment.

The gold standard method to acquire morphological information is to use Magnetic Resonance Imaging (MRI). MRI is a diagnostic imaging technique that is widely used to depict the anatomy and the dynamics of physiological processes happening in the body. For muscle studies in particular, a prolongation in T2 relaxation time between rest and intensive muscle activity has been reported to be useful for a quantitative evaluation of muscle activity [1]. This technique is referred as muscle functional MRI (mfMRI) and has been used in studies on lower back [3,4,5,6], lower limbs [7,8], upper limbs [9,10], shoulder [11], hip abductors [12] and plantar flexion movements [13]. It allowed the estimation of the activity of all muscles in the considered field of view with a single scan, showing particular advantage for the study of deeper muscles that have usually prohibitive access with other methods.

However, since a considerable time span is necessary between two T2-relaxation time measurements, the temporal resolution of mfMRI is low and therefore it is not possible to have reliable information about the activation dynamics of contracting muscles. Additionally, mfMRI has shown limitation in the evaluation of task at low level force [1], because of a lower activity threshold compared to other technique used to study muscle activity such as electromyography (EMG). EMG is a medical signal acquisition technique that read the potential variation caused by contracting muscles and it can be invasive (needle EMG) or non invasive (surface EMG). Usually surface EMG (sEMG) is collected with single or bipolar electrodes placed above the target muscles; however, the recent introduction of High Density sEMG (HD-sEMG) [14] allowed the collection of a significantly higher volume of data with the use of electrodes matrices placed above an area covering multiple muscles. Due to the higher number of electrodes however, the information is highly redundant and noisy and therefore a burdening post processing is usually needed.

Estimation of muscle activation using only sEMG was proposed and several works were presented with the use of HD-sEMG [15,16,17]. However, an important limitation of these studies relies on the fact that deep muscle activation cannot be estimated. Furthermore, these methods estimate the motor units’ activity ignoring morphological information about the person’s anatomy.

To cope with the limitations of mfMRI and sEMG, studies tried to merge the two techniques [5,9]. However, the outcomes were not always in agreement, and a direct relationship between increase in T2 value and in muscle activity was not always observable [1]. The reason for this discrepancy is alleged to reside in the nature of the information that is collected by the MRI and the sEMG, respectively. The information reflects the metabolic activity within the muscle for the MRI and records the neural electrical activity for the EMG. Thus, the need for a better merging of the two techniques to obtain significant information has emerged while overcoming the underlying conflict. Alternative hybrid MRI-EMG approaches in the literature are lacking and only a few works could be found [18,19]. However, these solutions required the use of discretized models and of heavy optimization which make them not suitable for clinical application due to the processing and computational time required.

To find a better symbiosis of the information provided by MRI and EMG, our group recently proposed an alternative methodology that provides an estimation of the forearm muscle activity. The method exploits the morphological information contained in the MRI scan to build an electrical lumped model of the conductive volume. Such model is then used to solve an inverse problem starting from the sEMG collected from a dense array of electrodes around the forearm [20,21]. The method, unlike previously proposed methods, provide an estimation of all muscles’ activation with a value for each muscle that lump its activity. With this paper, we evaluate the method on a group of participants performing different tasks involving different muscles, in order to observe how the method perform. An electromyographic dataset of four healthy subjects performing wrist and finger isometric tasks, has been studied. Since a direct validation of the estimated activities via needle EMG is impossible due to the difficulties and discomfort that it would entail for the subject, the performance was evaluated using specialized literature and quantifying the fitting of the sEMG with the projection of the estimated currents on the electrodes space. The study was conducted on the average muscles behaviour, therefore any time dynamics has been ignored and the root mean square (RMS) of the sEMG data was considered during each of the contraction tasks.

The results shows that, for four isometric contractions at different force level, our method is able to provide sets of muscles’ current that explain over 90% of the average sEMG information. The currents’ time profiles have a physiological explanation in most of the studied case and the method highlights subjective difference in the activation pattern for each subject given the same task. Furthermore, different level of force do not change the muscle activation profile in accordance to what is reported in the literature.

The details of the method in all its parts along with the description of the experimental setup is reported in the Material and Methods section. In particular, how the MRI and the EMG are collected and how the model is constructed from this information is described. The results are reported and discussed in the Results and Discussion section, respectively. Final comments and considerations about possible development and future application are then described in the Conclusion section.

## 2. Experimental Procedure

### 2.1. Subjects

Four healthy subjects (2 males and 2 females, average age = 27.3±2.3 years old) volunteered for this study. All the subjects gave their consent to take part to the experiment. The experimental protocol was approved by ethics committee of the University of Tokyo. None of the subject had any reported history of neuromuscular impairment at the time of the experiment.

### 2.2. MRI Acquisition

A T1-weighted MRI of the forearm has been acquired for all the subjects with a 3T Siemens Verio (Siemens, Germany) scanner at the National Center of Neurology and Psychiatry (Tokyo, Japan). The slice resolution was set to 0.2 × 0.2 mm while the resolution along the longitudinal axis i.e., slice thickness, has been set to 3 mm. Repetition time was 11 ms, Echo time was 4.92 ms and a 448×448 matrix has been considered. The forearm was held in supinated position for consistency with the setup during sEMG data. Vitamin E capsules were attached to bony reference point on the wrist and on the elbow as contrast markers for subsequent registration purposes.

### 2.3. Experimental Setup and HD-sEMG Acquisition

**Figure 1:**
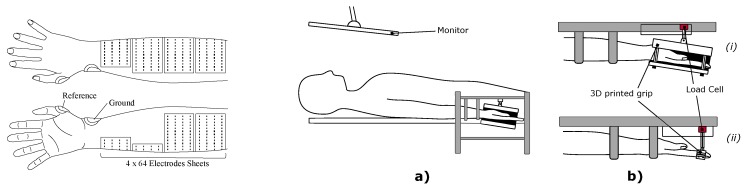
*Left*: Top and bottom view of electrodes positioning on the forearm. Four sheets of 64 electrodes were wrapped around the forearm in order to cover the maximum amount of its surface. A reference electrode was positioned on the metacarpophalangeal joint of the thumb. A ground electrode was sticked to the radial styloid. *Right*: Experimental setup. (**a**) Position of the subject during data acquisition. The subject was lying down facing a monitor through which a visual feedback was given of the applied force. The forearm was held in supine position, with the hand palm facing upward. (**b**) Force measuring structure for wrist movements (i) and for finger tasks (ii). The hand/finger was enclosed in a custom 3D printed structured coupled with a load cell to measure the force amplitude.

The experimental setup is depicted in Figure 1. The subject lay supine. A real time visual feedback of the exerted force was given with a monitor mounted over the subject’s face. The right forearm of the subject was then positioned on a custom made alloy frame that sustained the forearm and the hand horizontally aligned along the body. The wrist was in supinated position with the hand facing upwards in order to avoid crossing between the ulna and radius bone.

The hand was sustained with a 3D printed cage connected to force sensors in lateral and palmar position to measure the lateral and vertical forces, respectively. During the finger contractions the setup was slightly changed. The target finger was constrained inside an additional 3D printed structure connected to another force sensor to measure the exerted force along the direction normal to the surface of the finger tip (Figure 1a,b). Forces were measured with an Arduino board sampling at 10 Hz.

Each subject had to perform five different isometric tasks at two levels of force, 25% and 50% of maximum voluntary contraction (MVC). The isometric tasks executed were the following:

1. Middle finger metacarpophalangeal joint (MCP) extension
2. Wrist flexion
3. Wrist extension
4. Ulnar deviation
5. Index finger MCP extension

Each of the listed tasks was repeated 15 times. Each repetition consisted of a 2 s force raising, 5 s force holding, 2 s release and 15 s of rest. For the purpose of this analysis, the first two and the last two contractions has not been considered to avoid effect of adaptation and fatigue, therefore all the analysis was conducted on 11 repetitions. An additional contraction at maximum force has been done prior to each type of task, in order to set the MVC force value.

Since the main responsible for index finger MCP extension, extensor indicis muscle, is located in a more distal section of the forearm compared to the muscles involved in the other considered task, in this work, the index finger MCP extension was not considered.

The HD-sEMG has been acquired with four custom designed electrodes’ sheets wrapped around the forearm at different lengths around the forearm, so to cover as much of it as possible. The forearm skin was cleaned with alcohol previous to attach the HD-sEMG sheets. Each electrodes’ sheet had 16×4 electrodes and a total of 256 channels were acquired on the forearm. Two different sized sheets were used depending on the size of the subject’s forearm: a shorter sheet of 195 mm ×52 mm size with 10 mm ×19.5 mm inter-electrode spacing, and a longer sheet of 270 mm ×52 mm size with 15 mm ×19.5 mm inter-electrode spacing. For the purpose of this work a single row of electrode properly selected around the forearm was considered.

The signal was acquired with a RHD2000 Evaluation System (Intan Technology, City, State USA) with a 2500 Hz sampling frequency with no prior analog filtering. Additional adhesive electrodes were placed on the radial styloid and at the metacarpophalangeal joint level of the thumb as, respectively, ground and reference (Figure 1).

The position of the electrodes and of the anatomical references marked in the MRI were recorded with a Polhemus Fastrak 3D digitizer system (Polhemus, Colchester, VT, USA). The forearm was susequently wrapped with an elastic bandage to keep the electrodes sheet adherent to the skin.

The sEMG measurements were collected for all tasks in a single session, a few days after the MRI acquisition.

## 3. Proposed Method

We propose a method that allows the estimation of the muscle activation pattern from a selected MRI slice and the EMG signals collected from electrodes around the selected section. The conductive volume and the interaction between the different tissues is described with a lumped parameter model that can be represented with a graph.

The method simplify the solution of the inverse problem for the identification and the quantification of the activity of muscles involved. The conductive properties in a selected section of the arm is described with a resisitive network connecting the different tissue elements and the electrodes. The MRI section is chosen in order to include the muscles responsible for the target movements.

Muscles are identified with a segmentation step and, along with the electrodes, identify the nodes of a graph which edges are defined based on the anatomy observable from the MRI slice. The model is then solved assuming the muscles as current generators placed on muscle nodes to obtain an estimation of muscles activity.

**Figure 2:**
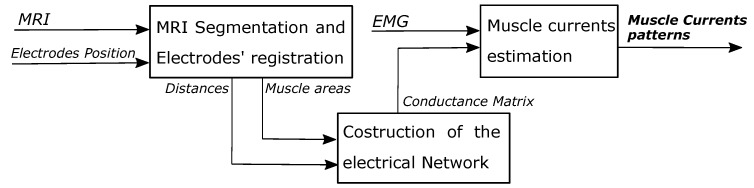
Diagram of the proposed approach. Three main parts can be identified, MRI segmentation and electrodes’ registration, Construction of the electrode network and muscle currents estimation. From the left, the MRI segmentation and electrodes’ registration block process the information in the MRI and about the electrodes’ position to estimate the distance between the elements (muscles and electrodes) in the MRI and the muscles’ cross section areas. This information is then used to create and weight the edges of a graph describing the interaction between the nodes representing muscles and electrodes. In the Muscle current estimation block, the EMG is exploited to obtain an estimation of the muscle activation patterns solving an inverse problem using the conductance matrix that describes the graph and the related electrical network.

The model construction process can be separated into three main sections (Figure 2) which will be explained in detail within the next subsections:

1. MRI segmentation and electrodes’ registration
2. Construction of the electrical Network
3. Muscle currents estimation.

### 3.1. MRI Segmentation and Electrodes Registration

To discriminate the different tissues and the different muscles within the selected MRI slice, a segmentation process is necessary. Segmentation is the imaging process through which the different anatomical structures are identified on a diagnostic image. In the forearm it is possible to mainly identify four types of tissues: bone, skin, fat, and muscular tissue.

**Figure 3:**
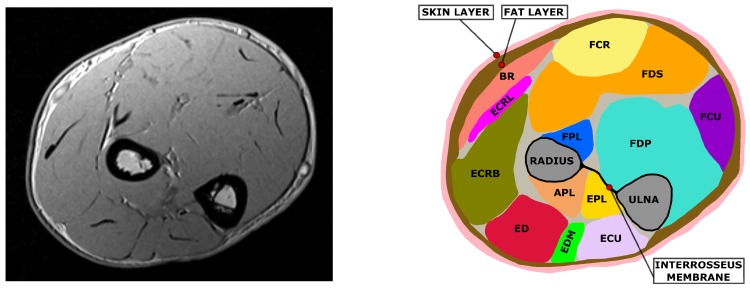
Example of segmentation on an MRI slice. On the left the original MRI is depicted, on the right its segmentation is reported. Muscles are represented with different colors, the radius and ulna bones are reported in gray. It is possible to observe the interosseus membrane connecting ulna and radius and dividing the posterior and the anterior side muscles. Skin and fat layers are colored respectively in pink and brown.

While the skin, fat and bones can be easily identified due to their different contrast in the T1-weighted MRI image, muscle tissue needs to be subdivided into the different muscles that compose it. The forearm contains about 20 muscles which are responsible for forearm pronation/supination, wrist radial/ulnar deviation and flexion/extension, and for finger flexion/extension. It is possible to roughly divide the forearm’s muscles into two groups based on their function: extensors along the posterior side and flexors on the anterior side. Additionally, extensor and flexors can be further divided into superficial and deep muscles depending on their positioning in the cross-section. Muscles delineate complex paths along the forearm. Cross sections at different longitudinal coordinate of the forearm can therefore be identified as different muscle sets. The segmentation process allows for the identification and definition of the muscle’s position, cross sectional areas, and boundaries (Figure 3).

For all subjects a single slice was selected. The selected slice contains for every subject the same set of muscles, which were identified using the imaging software ImageJ [22]. The considered set of muscles was the following: Abductor Pollicis Longus (APL), Extensor Pollicis Longus (EPL), Brachioradialis (BR), Extensor Digitorum (ED), Extensor Carpi Radialis Longus (ECRL), Extensor Carpi Radialis Brevis (ECRB), Extensor Carpi Ulnaris (ECU), Extensor Digiti Minimi (EDM), Flexor Digitorum Profundis (FDP), Flexor Digitorum Superficialis (FDS), Flexor Carpi Ulnaris (FCU), Flexor Carpi Radialis (FCR).

The skin layer has been ignored for the purpose of this work and its thickness has been included into fat layer thickness. Fat layer thickness is estimated as the average value of electrode-muscle distance along the arm circumference.

**Figure 4:**
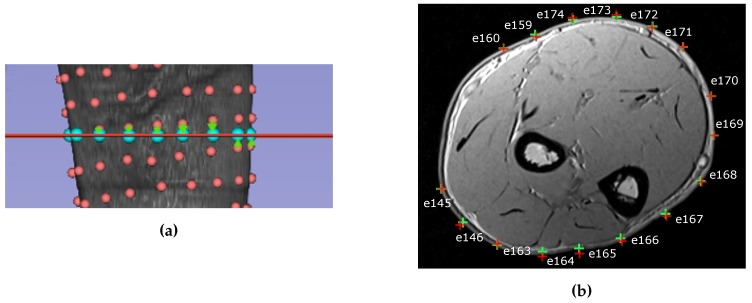
(**a**) The projection of the electrodes closest to the selected slice plane. The real electrodes position is indicated with a red sphere, the MRI plane is indicated with a red line and the projected position is indicated with a light blue sphere. The projection is depicted with a green arrow. (**b**) Section of the MRI with the position and the number of the electrodes. The projection of the electrode 3D position on the 2D slice is represented with a red cross. Such points are further projected on the skin (green cross) to find the closest position on the forearm surface.

To correctly place the electrodes on the MRI volume a registration step has been done prior to the creation of the model, since electrodes’ coordinates and MRI anatomy have been acquired with different reference frames. Registration estimates the optimal transformation to match the coordinate system of the electrodes to the coordinate system of the MRI. The registration has been done using 3D slicer [23,24], minimizing the distance between the anatomical markers on the MRI and the markers acquired with the electrodes coordinates using the digitizer. The electrodes around the arm that were closest to the chosen slice were selected and their 2D projection on the MRI section was calculated (Figure 4a). The position of the electrodes on the slice was set as the closest point on the skin of the projected electrodes (Figure 4b). The mean and the maximum distance error made during electrodes’ projections steps are reported in Table 1 for all subjects. On the upper rows the errors for the projection on the selected slice are reported while on lower rows the errors on the projection on the skin surface are reported. On average the error during the projection on the selected slice is between 3 and 4.2 mm, with maximum values ranging from 7.5 to 11 mm. The distance errors on the projection on the skin surface was lower, with an average error smaller than 1 mm for all subjects and maximum values going from 1.5 to 3 mm.

Once muscles and electrodes are correctly positioned and identified on the MRI slice plane, it is possible to measure the relative distance between muscles and electrodes. In particular, it is possible to identify three type of distance:

- electrode-electrode (e-e) distance
- muscle- electrode (m-e) distance
- muscle-muscle (m-m) distance

Distances have been calculated from the centroid of the segmented cross sectional area for muscles. Electrodes’ distances were calculated from the projection of the electrode position on the skin. In an MRI slice it is possible to identify $n_{m}$ muscles and $n_{e}$ electrodes on the skin surface.

### 3.2. Construction of the Electrical Network

**Figure 5:**
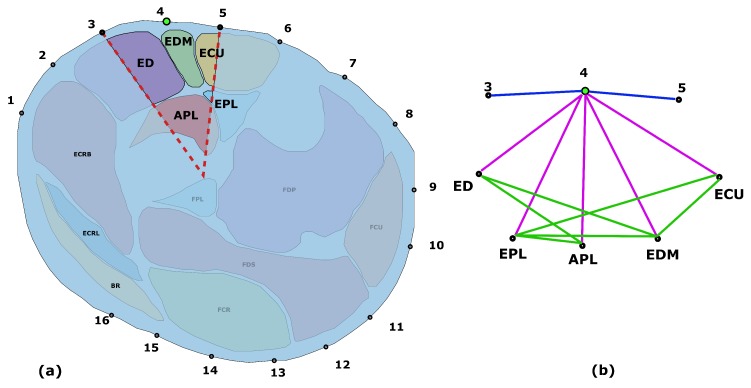
Example of one step of the graph creation. (**a**) Sector belonging to one of the electrodes, indicated with a green dot. The muscles that are included (ED, EDM, ECU, APL, and EPL) are highlighted with the area that is accounted for. (**b**) Graph created from the section considered in (a). e-e edges are indicated in blue, e-m edges are indicated in purple and m-m edges are indicated with green lines.

The anatomical information extracted with the segmentation can be described with a topology. A topology can define the connections between the different elements identified in the segmentation. One of the most common way to describe a topology is a graph, a mathematical discrete structure constructed with a set of nodes and a set of edges. A node represent an object of the structure that carry a different meaning depending on the application. Edges describe a relationship between a pair of nodes. To describe the anatomical information contained in the MRI slice it is possible to model muscles and electrodes as nodes of a graph, with edges that describe connections that are defined with a set of custom rules based on several assumptions and prior anatomical information. The rules used in this work are listed as follows:

i Each electrode defines a sector of the forearm section bounded by the lines going from the centroid of the MRI section to the two adjacent electrodes as shown in Figure 5.
ii All muscles included in a sector with a fraction of their area greater than 5% of their cross sectional area are connected to the electrode that defines that sector.
iii If between the fraction of the muscle in a sector and the electrode defining that sector there is a bone structure, that edge is not considered.
iv In general a muscle node is connected to another muscle node only if they are adjacent on the segmented image. Exception are considered to take into account specific anatomical structure that are known to alter the currents flow in the volume. In particular the effect of the interosseus membrane between the ulna and the radius has been considered.
v The fat layer is assumed to be of constant thickness around the section circumference. Such thickness is set to be the average obtained from the segmentation and it includes the skin thickness.
vi Since it is an electrical network and additional node has to be added to model the ground.

This set of rules allows the definition of a graph with *N* nodes and *E* edges that specifically describes the anatomy of the subject and the electrodes setup for a specific cross section of the forearm. Each edge is weighted based on the nodes pair $\left(n_{1},n_{2}\right)$ that defines it. The weight is set to be the electric conductivity $G_{n_{1}n_{2}}$ between the nodes pair obtained as

(1) $$G_{n_{1}n_{2}}=g\cdot r\left(n_{1},n_{2}\right)$$

where *g* is the conductance of the tissue interposed between the 2 nodes and $r(n_{1},n_{2})$ is the distance between the nodes $n_{1}$ and $n_{2}$. The conductance for the considered tissues (i.e., muscle, fat and skin) are those reported by Lowery et al. in [25]: $g_{skin}=4.3\cdot 10^{-1}$*S/m* for the skin, $g_{fat}=0.04$*S/m* for the fat and $g_{muscle}=0.09$*S/m* for muscle along the transverse direction.

The nature of the node pair in the edges define the type of conductance that is chosen. As described for the measured distance, it is possible to identify electrode-electrode(e-e), muscle-muscle(m-m) and electrode-muscle(e-m) type of edges. For (e-e) and (m-m) edges, they can be directly weighted using Equation (1), assuming that the current between two adjacent electrodes flow through the skin and that the current between two muscles flow through only muscle tissue:

(2) $$\begin{array}{c} G_{ee}=g_{skin}\cdot d_{ee}\\ G_{mm}=g_{muscle}\cdot d_{mm}. \end{array}$$

In the case of (e–m) edges the conductance to weight the edge has been set to the series of two resistive elements in order to consider that current flow from muscle to electrode passing through muscle and fat tissue:

(3) $$G_{em}=\frac{G_{fat}\cdot g_{muscle}\left(d_{em}-D_{fat}\right)}{G_{fat}+g_{muscle}\left(d_{em}-D_{fat}\right)}.$$

To consider the effect of partial inclusion in the sector, the conductance of the (e-m) edges were weighted with the normalized area:

(4) $${\hat{G}}_{em}=G_{em}*\left(\frac{A_{em}}{A_{m}}\right)$$

where $A_{em}$ indicates the area of the muscle *m* included in the sector defined by the electrode *e*, and $A_{m}$ is the total area of the muscle *m*.

Each of the muscle nodes was modelled as a current generator to represent the active muscle behaviour. Finally, all the nodes were connected to the ground node with a shunt resistance. The shunt resistance has been set to $D_{skin}\cdot G_{el-el}$ and $D_{muscle-bone}\cdot G_{muscle}$, respectively for electrode nodes and muscle nodes, where $D_{skin}$ is the average distance between electrodes and $D_{muscle-bone}$ is the average distance between the centroid of muscles and the closest bone (ulna or tibia).

The electrical model so built can be described exploiting the principles of graph theory. Under the assumption of no external power injection and in static condition the current of the generators can be described with the following relationship [26]:

(5) $$I=(L_{R}+G)V$$

where $I\in R^{N\times 1}$ is the vector of currents, $V\in R^{N\times 1}$ is the vector of node potentials, $L_{R}\in R^{N\times N}$ is the conductance matrix and $G\in R^{N\times N}$ is the shunt conductance matrix. If G has at least one non-negative element, Equation (5) can be inverted:

(6) $$V={\left(L_{R}+G\right)}^{-1}I=FI$$

where $F={\left(L_{R}+G\right)}^{-1}$ is the matrix describing the system. Using Equation (6) it is possible to estimate the potential of the nodes with respect to the reference value, which is usually set to $V_{0}=0$V.

#### 3.2.1. Muscle Currents Estimation

Using the built model, the muscle activities are estimated solving an inverse problem. Equations (5) and (6) describe the linear relationship that relate the currents and the voltages in the network. Equation (5) describes the relationship from the potentials and the currents on the node. Since we are only interested in how the muscles’ current are reflected on the electrodes potentials it is possible to further simplify the problem reducing the dimension of the system matrix, i.e., selecting the row corresponding to electrodes nodes and columns corresponding to muscle nodes from the matrix *F*. The resulting matrix is a matrix $\tilde{F}\in R^{n_{e}\times n_{m}}$ where the $i^{th}$ column is the potential that would be present on the $n_{e}$ electrodes if the $i^{th}$ muscle is activated with unitary current.

With this notation it is possible to describe the transformation from muscles currents to electrodes’ potentials as follows

(7) $$V_{e}=\tilde{F}I_{m}$$

where $V_{e}$ is the $n_{e}\times 1$ electrode potential vector and $I_{m}$ is the $n_{m}\times 1$ muscle current vector.

Since the number of electrodes is always higher than the number of muscles, the problem is over-determined. A minimum norm approach would therefore lead to unstable solutions. To find a unique solution, a regularization is needed. We assumed that the voltage measured at the sEMG is caused mainly by the activities of big muscles, therefore the regularization is penalizing the activation of muscles with small cross sectional area. The resulting estimated muscles current $\hat{I_{m}}$ has been therefore calculated as follows:

(8) $$\hat{I_{m}}={\tilde{F}}^{T}{\left(\tilde{F}{\tilde{F}}^{T}+\lambda I\right)}^{-1}V_{e}$$

where λ is a regularization vector that has been set to the inverse of the cross sectional area of all the muscles.

#### 3.2.2. Validation

A direct evaluation of forearm’s muscle activity would entail the use of invasive probe directly inserted on each of the considered muscles. This is obviously practically impossible because of the pain and the discomfort that it would cause to the subject. Therefore, to validate the results indirect methods must be used. In this work we considered two criteria, one qualitatively based on functional prior knowledge and the other quantitatively measuring the fitting on the measured sEMG data on the electrodes’ space. To qualitatively evaluate the results from an anatomical point of view, the muscles involved in the examined motor task has been compared to those reported in the literature. In particular the muscles involved in each of the movements are the following:

− Middle finger extension: ED, EDM
− Wrist flexion: FCU, FCR, APL, EDM
− Wrist extension: ECU, ECRL, ECRB, ED
− Ulnar deviation: FCR, FCU, ECR, ECU

The method was evaluated comparing the estimated active muscles with those reported as main responsible by specialized literature [27,28]. A quantitative performance evaluation of the method is conducted comparing the projection of the estimated muscle on the electrode space with the measured sEMG, to express the amount of the sEMG information that is explained by the estimated activity. The Goodness of Fit (GOF) is evaluated using the Normalized Root Mean Square Error (NRMSE) defined as follows:

(9) $$GOF=1-NRMSE=\frac{RMSE}{max-min}.$$

With this definition, a value of GOF close to 1 indicates a higher fitting on the experimental sEMG data, with 1 indicating a perfect fit. Lower values indicates lower fitting performance.

## 4. Results

**Figure 6:**
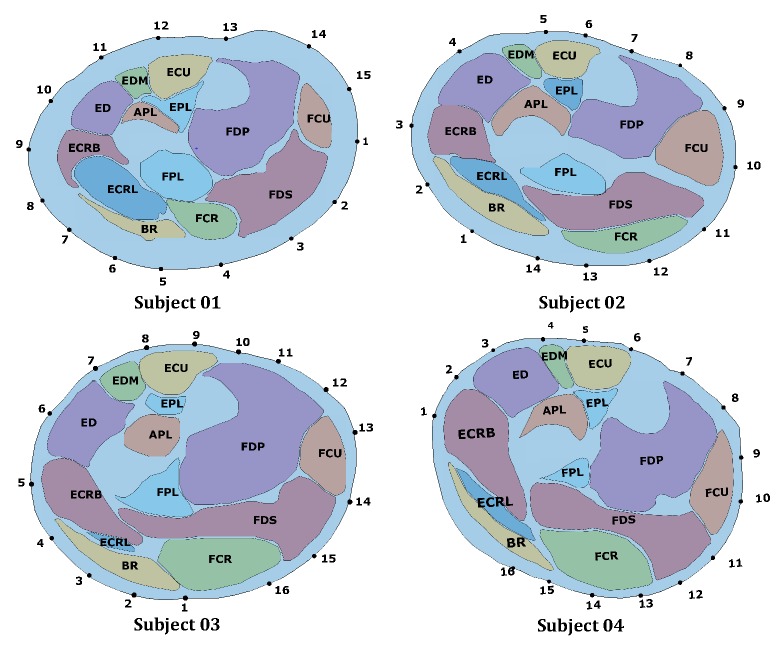
Results of the segmentation on muscles and registration of electrodes position on the 2D image plane of the MRI slice. In each MRI slice the following muscles can be identified: Abductor Pollicis Longus (APL), Extensor Pollicis Longus (EPL), Brachioradialis (BR), Extensor Digitorum (ED), Extensor Carpi Radialis Longus (ECRL), Extensor Carpi Radialis Brevis (ECRB), Extensor Carpi Ulnaris (ECU), Extensor Digiti Minimi (EDM), Flexor Digitorum Profundis (FDP), Flexor Digitorum Superficialis (FDS), Flexor Carpi Ulnaris (FCU), Flexor Carpi Radialis (FCR).

The results of the segmentation and registration process are represented in Figure 6. Each muscle is represented with a different color and the projection of the electrodes on the skin is represented with a dot and a number indicating the sequential order of the electrodes.

**Figure 7:**
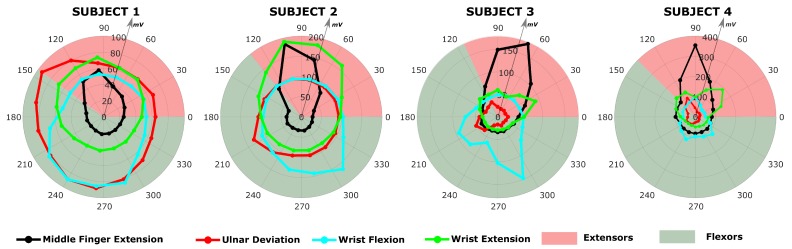
sEMG RMS profile for the four considered tasks at 50% MVC for all the subjects. Each isometric task profile is represented with a different color with a circle indicating the local value measured on the electrode. The electrodes are assumed to be equally distributed around the circumference of the cross section of the forearm. The posterior and anterior side, corresponding to the sectors where extensor and flexors muscles are located, are represented with different background color.

The RMS profile of the sEMG for the studied isometric tasks at 50% of the MVC are depicted with polar plots in Figure 7. Each tasks is shown with different line color. For the sake of clarity the polar plots were created assuming that the arm section is circular and that it is divided into $n_{e}+1$ sectors by $n_{e}$ electrodes arranged with equal space between each other. The value of the RMS voltage is represented with a circle on the line. Each subject has a different number of electrodes depending on the size of the forearm. To better identify the position of the electrodes, the portion of the cross section area with extensor muscles (posterior side) and flexor muscles (anterior side) are depicted with a different background color on the section, respectively with in red and green.

The first two subjects have similar RMS values with similar RMS sEMG profiles along the circumference, while subject 3 and 4 have a higher RMS value for the middle finger extension. For each of the tasks it is possible to observe a subjective sEMG profile around the arm.

**Figure 8:**
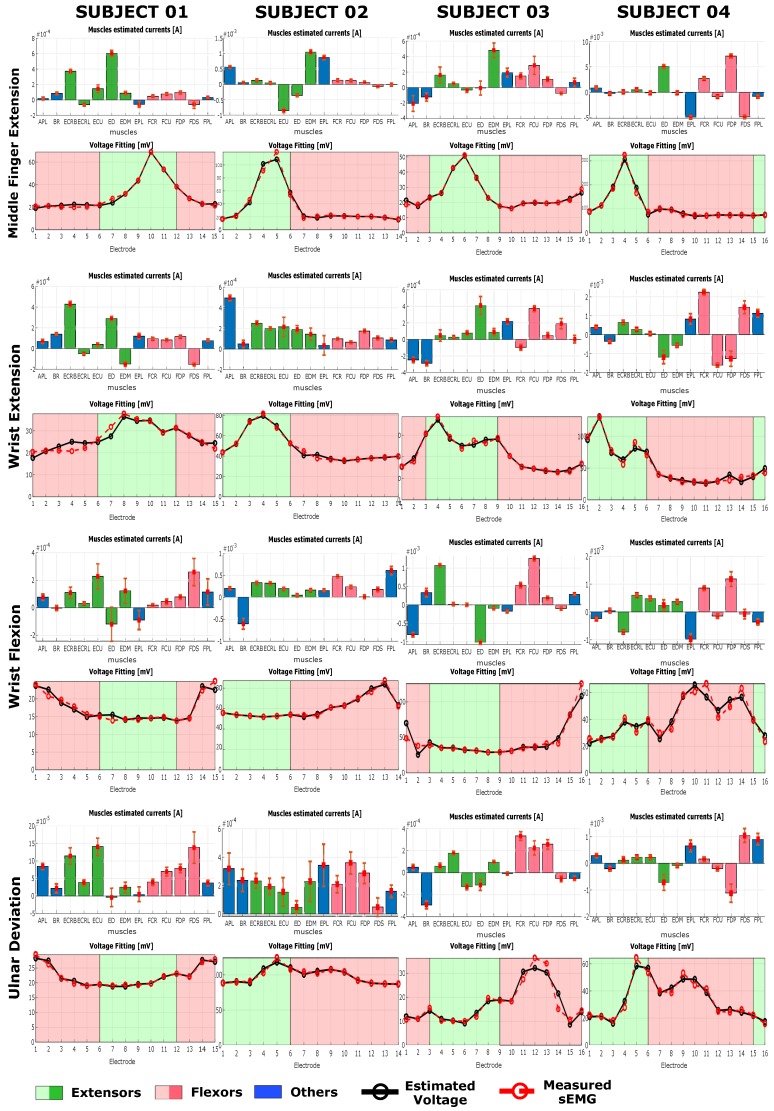
Estimated currents and voltage fitting on the electrodes space for all the tasks considered at 25% of MVC. Each bar represents the average current estimated for a muscle for 11 repetitions. The bar is coloured in green for extensors and in red for flexors. Muscles that are not mainly involved in the considered movements according to anatomy are colored in blue. On the electrodes space, the measured sEMG is represented with a red dotted line, whereas one example of the estimated value is represented with a solid black line. The background color is green for electrodes over the posterior side of the forearm (extensors) and is red for electrodes over the anterior side of the forearm (flexors). For further information on the meaning of the abbreviation of the muscles and their position refer to Figure 6.

**Figure 9:**
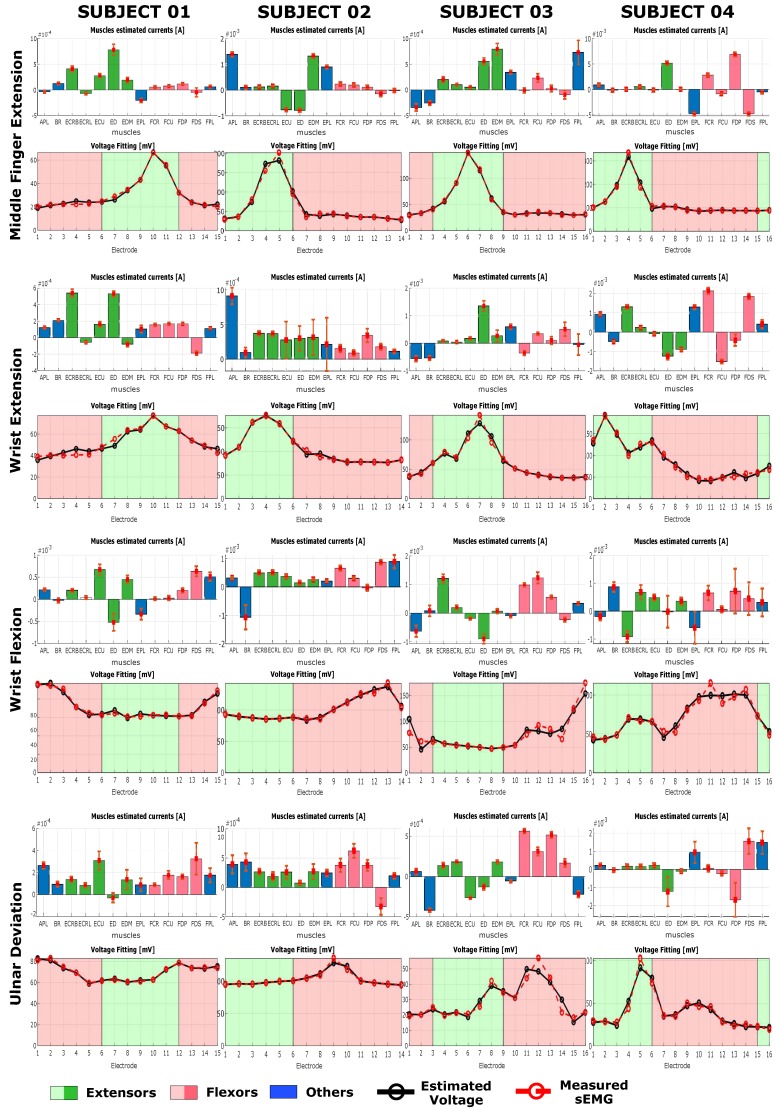
Estimated currents and voltage fitting on the electrodes space for all the tasks considered at 50% of MVC. Each bar represents the average current estimated for a muscle for 11 repetitions. The bar is coloured in green for extensors and in red for flexors. Muscles that are not mainly involved in the considered movements according to anatomy are colored in blue. On the electrodes space, the measured sEMG is represented with a red dotted line, whereas one example of the estimated value is represented with a solid black line. The background color is green for electrodes over the posterior side of the forearm (extensors) and is red for electrodes over the anterior side of the forearm (flexors). For further information on the meaning of the abbreviation of the muscles and their position refer to Figure 6.

The results of the current estimation procedure and the relative voltages reprojected on the electrodes’ space are reported in Figure 8 and in Figure 9 for tasks at 25% MVC and 50% MVC, respectively. For each subject and given task, the top graph represents the estimated currents generated by each of the muscle node, while the lower graph represents the reprojected voltage on the electrode space compared to the measured sEMG. The bar on the top graph represents the average current estimated for the generators on each muscle node for all the 11 contractions considered and are coloured based on their function. On the lower plot, the black dotted line represents the projection of the estimated currents on the voltage space whereas the measured sEMG is represented with a dashed red line. The background color is represented with red for electrodes on the anterior side, i.e., electrodes over flexor muscles, and green for electrodes on the posterior side, i.e., electrodes over extensor muscles. The number of electrodes is different for each subject and the number of electrodes of the anterior or posterior side changes depending on the subject.

A quantitative evaluation of the voltage fitting onto the electrodes space is reported with GOF values in Table 2 with the average value and the standard deviations over the 11 tasks considered.

## 5. Discussion

Observing the plots in Figure 8 and Figure 9 for each of the tasks, the inter-subject and inter-task differences can be seen [29,30,31]. For a given task, however, the group of muscles involved is not significantly changing between subjects, but it is possible to observe subjective characteristics in muscle activation. No significant changes can be observed in the activation pattern between different force level.

Each of the tasks shows specific muscle activation pattern that are in most of the case in agreement with what is reported in the anatomical literature. Each of the subject shows a subjective variation on the task activation pattern, especially for middle finger extension, (MFE), wrist extension (WE) and wrist flexion (WF) (Figure 8 and Figure 9 ). Ulnar deviation (UD) shows a higher variability among subjects in both the estimated muscle pattern and in the input sEMG. UD could have a marked variability between subject depending upon the prono-supination and wrist flexion extension angle at which the subject have their neutral position. For a slightly flexed wrist, activation of the flexor muscles will be higher during ulnar deviation when compared to slightly extended wrist.

During middle finger extension (MFE), for all subjects and in both force conditions a clear peak activity was registered on one or two extensor muscles, and for all subjects there was at least one muscle active among ED and EDM, indicated in the literature as major responsible for that task. In particular, looking at both Figure 8 and Figure 9, for subject 1 there is a peak on ED with a lower peak on ECRB, on subject 2 there are peaks on EDM and EPL and a negative peak also on ECU, on subject 3 there is a clear peak on EDM, while on subject 4 the peaks are on ED and EPL, with this last one negative. During WE it is possible to observe a general activation of extensor muscles on all subject, with subject 1 showing a peak activation of ECRL and ED, subjects 2 showing a more homogenous activation among all extensors and subject 3 and 4 showing a sparse activation with clear peaks on, respectively ED for subject 3 and on ED and EDM for subject 4. On subject 2 a peak on APL can be observed as well. APL is not actively involved in the extension task but its activation can be due to the fact that the subject tried to spread the hand fingers, including the thumb, during the extension of the wrist. The fact that the activation pattern for subject 2, unlike the other subjects, is homogeneous among all extensors might indicates that the subject extended the fingers while trying to extend the wrist.

During WF, it is possible to observe a peak activation on FDS on subject 1, peaks on FCR, FCU, and FPL on subject 2, peaks on FCR and FCU for subject 3 while for subject 4 there is only a peak on FCR and FPD. These results are in accordance to the anatomical knowledge, and each of the subject uses a different muscle among the possible ones to perform the same task. Subject 2 shows an additional significant activation of BR, indicating that, probably, during the flexion of the wrist the subject also tried to flex the forearm around the elbow joint.

For ulnar deviation (UD) results are according to anatomical knowledge in the case of subject 1, 2, and 3. In fact it is possible to observe peaks on either one or both of ECU and FCU. Subject 4 presents an activation on ED, EPL and FDP, while muscles that are indicated as main responsible for UD i.e., ECU, FCU, FCR, ECRL, ECRB, show a lower activation level. Observing the measured sEMG of UD for subject 4, it shows a different shape compared to the other subjects, indicating that the difference might be due to a subjective muscle activation pattern characteristic of that subject.

Observing both the estimated muscle activation profile (Figure 8 and Figure 9) and the measured sEMG profile (Figure 7) around the forearm, each of the subjects perform each of the task in a characteristic way. Observing the input sEMG profiles it can be noted that for a chosen task there are important differences among subjects. The difference in the morphology of the forearm could explain different muscle patterns estimation. In the case of MFE, observing the sEMG profiles in Figure 7, it can be noticed that a similar profile shape among subjects is shared, where a single peak in the posterior side of the forearm muscles is present. For subject 2 the activation peak is shifted more toward the muscles on the anterior side, which might justify higher peaks on EDM and EPL. This means that all subjects performed the MFE in approximately the same way, activating either the same muscles or group of muscles around the same section of the forearm. On the other hand, a different relative activation is present, based on how the neural system of each subject adapted to perform that specific task. In the case of WE and WF, subject 1 and 2 show a measured voltage with a smoother profile around the arm, whereas subject 3 and subject 4 present a more irregular profile with a biphasic behaviour. An even bigger difference can be observed for UD, where subject 1 and 2 present a rather smooth and flat sEMG with a peak on the flexor side of the arm, while subject 3 and 4 presents clear peaks. In particular subject 4 showed a biphashic sEMG profile with voltage peaks measured around electrodes 5 and 10 (Figure 8 and Figure 9). Since UD, due to the nature of the movement, involves both extensor and flexor muscles, it can be seen that in general the peaks in the sEMG profiles are around the boundary area between extensor and flexors. However, each of the subjects present a characteristic shape, indicating that for this movement the muscle pattern might change significantly between subjects. The reason behind this apparent difference in the sEMG profile might be the position of the forearm with respect to the neutral position of each subject during the experiment. The supination angle of the forearm during the data collection might not have been perfectly horizontal causing a different activation of muscles.

Comparing the muscle signal patterns between 25% and 50% MVC conditions (Figure 8 and Figure 9), they remain almost unchanged. This means that each subject maintain their specific muscle activation strategy among different force conditions. Each of the subjects therefore keeps activating the same muscles with the same strategy at different force levels, increasing the overall muscle activity of the involved muscles, without using other muscles or changing activation pattern. The method is therefore robust to different force conditions for the same task. However, it is important to notice that the force conditions and the choice of ignoring the first and the last repetition were meant to minimize the risk of having adaptation and fatigue effects. In fact, in case of adaptation and fatigue conditions the activated muscle pattern might undergo changes due to a varying muscle recruitment pattern from the CNS [32,33,34,35].

The projection of the estimated current on the electrodes potential space fits the measured sEMG with a GOF over 90% for almost all of the task considered in both force conditions (Table 2). Thus, the currents estimated with the proposed model are able to explain on average more than 90% of the information enclosed in the RMS of the measured sEMG. Exceptions are WE for subject 1 at 25% MVC and WF and UD at 50%MVC for subject4, where GOF is slightly under 90%.

In different cases it is possible to observe a simultaneous activation of both agonistic and antagonistic muscles, i.e., flexors and extensors, thus co-contraction is happening. Furthermore, it is worth noting that in several cases, such as MFE, WE, UD for subject 4, MFE and WE for subject 1 or WE, WF, and UD for subject 3, currents with opposite signals are estimated for antagonistic muscles. Therefore, we hypothesised that the sign of the current is indicating the direction of the power exerted by the muscles. That would mean that the sign of the current is giving mechanical information of the system, such as the type of contraction of the muscles, which can be concentric or eccentric. The estimated voltages at the muscles nodes were all positive, therefore the sign of the electrical power of the network was defined by the sign of the currents. This is however, does not seem to be valid in general, probably because of the fact that for this work only an average behaviour has been considered. A study on the time series estimation of muscle pattern would help supporting this hypothesis and it will be further considered in the future.

Overall the results show that, for a chosen task, a small signal variance is estimated among tasks repetition. Thus, on average, each of the task has been performed in the same manner by the subject. However, such as for UD at 50% MVC for subject 2 or WF at 50% MVC for subject 4, the estimated patterns show a higher variability, indicating that at each of the repetition significant differences in the contraction were happening.

The number of electrodes was different for all the subjects because of the different circumferences of each of the subject’s forearms. The electrode sheets were therefore able to cover a different amount of the forearm circumference as it can be observed in Figure 6. Subject 1 and subject 3 show a uniform coverage of the forearm surface with 15 and 16 electrodes respectively. Subject 2 and subject 4 present regions that are not covered with electrodes. In particular, for subject 2 the density of electrodes is lower over the extensor side of the forearm, while for subject 4 there were fewer than two electrodes directly over BR, ECRL, and ECRB. For subject 2, the lower electrode density over the extensor side of the forearm might be responsible for the higher activation of APL observed during WE. The fact that only one electrode is collecting the information above ED and APL might have mislead the inverse algorithm in the estimation of the activation pattern. On subject 4, the width of the area not covered with electrodes is bigger than that of other subjects. In particular, between the electrodes 1 and 16, no electrodes is directly over ECRL. This does not seem to influence too much the estimation of the results for MFE, WF and UD, since, observing Figure 8 and Figure 9, the sEMG values on electrodes 1 and 16 are low and of similar values. Since anatomically BR, ECRB and ECRL are not involved in these tasks, we can assume that the sEMG values in the electrode missing would be similar to that measured on electrodes 1 and 16. However, in the case of WE, ECRB and ECRL are muscles involved in the task. Observing the sEMG values on electrodes 1 and 16, they show a different amplitude. Therefore, it may be presumed that between electrode 1 and 16 the sEMG value is not low. As a consequence, the lack of electrodes in that forearm portion might have caused a loss of information that influenced the muscle pattern estimation for WE.

Finally, it is important to notice that in this work, the role of the skin conductance was ignored. Skin is known to have a strong low-pass filtering effect on the sEMG signal. Since only the average behaviour was considered, we think that for the purpose of this paper, this did not have a big influence on the results. However, it is important to notice that it might have an influence on the general amplitude of the estimated currents, since the conductivity value of the skin is significantly lower than that of muscles and of fat tissue. For this reason, the value of the single muscle current cannot be considered a precise estimation of the muscle activity. However, the relative activation among muscles remain unvaried in the estimation process. With this work, we rather want to show new simple way to estimate the activation pattern of a set of muscles rather than focusing on the single muscles.

## 6. Conclusions

We presented a novel method for the estimation of muscle activity. The activity was quantified using the muscle current estimated in an electrical system created from subject specific MRI images. The model is able to provide an estimation of the relative activation for a set of muscles identified within an MRI section of the forearm, through the construction of a purely resistive electrical model of the conductive volume depicted in the slice. The results highlighted some interesting properties of the method in the estimation of subject muscle activation patterns for potential rehabilitation applications. For the same task the estimated pattern were similar among subjects, but subjective activation features can still be observed among subjects, showing that the proposed method is able to capture anatomically valid muscle activation maintaining a subjectivity in the results that allows a comparison between different subjects. The input sEMG profiles changed with subject, reflecting the fact that each of the subject has a personal signature in the activation pattern, even if each of the single muscle maintain its properties and role. The proposed method is able to cope with this variability rearranging the muscle activation pattern to explain the input sEMG with a valid current pattern. Furthermore, the muscle patterns are not changing between different force levels, indicating that changes in the signal amplitude are not perturbing the estimated pattern. The estimated currents are able to explain over 90% of the input sEMG and the results have an anatomical meaning based on specialized literature and physiological consideration. The number of electrodes and their distribution might have had an influence in the estimation results. For two of the subjects the electrode density was lower in particular sections and this might have lead to different interpretation of the sEMG in the solution of the inverse problem.

With this work we wanted to introduce a novel idea for solving the problem of estimating deep muscle activities. We used a simple electrical model, providing results obtained using a dataset acquired from healthy subjects. The proposed model inherently includes information about each of the subject inner morphology described in a simple and intuitive way. Furthermore, the rules defined for the model construction and for the estimation can be easily automatized making the translation of the presented logic into a software routine with a low number of parameters.

The results shows that, for most of the task considered, the estimated muscle patterns are anatomically plausible. For simple static tasks, such as MFE, the estimated pattern, even if with a clear difference among subjects, provides an activation pattern that matches what is reported in the literature. In case of WE and WF, most of the case considered are anatomically consistent. However, some isolated results are uncertain. For UD, subject 1 and 2 shows valid results, while subject 4 shows different results, most likely due to a different sEMG profile around the arm, which indicate a different overall muscle activation, or an insufficient number of electrodes.

Several aspects of the model can still be improved, starting from the modeling to the estimation algorithm. In this work, we rather wanted to focus on the presentation of a different approach to exploits both information from MRI and sEMG to build a model able to solve the problem of muscle activity estimation, with a validation on a pool of healthy subject and with different movements in order to assess the performance that can be achieved with a simple and intuitive modeling. The impossibility to have a ground truth remain a challenging part for the validation of the method and it will be addressed in subsequent works with the aid of tools such as musculoskeletal modeling and simulations, even if the strong subjectivity of the muscle pattern activation is a point that makes the problem of a solid validation very challenging. A possible solution would be the use of ultrasound imaging during the execution of tasks. Using the ultrasounds it would be in fact possible to capture the thickness changes in all muscles, including deep ones. The thickness of muscles is related to its activation [36,37] providing a direct mean to validate the results. The RMS of the sEMG during contraction was considered for this work in order to give an average estimation of the behaviour of the muscles during the selected tasks. The introduction of the study on the time dimension would allow observing the contraction strategies of the muscles during the task execution. Furthermore, the subject pool was limited to four participants in this work. All these issues will be addressed in future developments of the proposed method.

We believe that this method can be a valid way for overcoming the limitations of the mfMRI, allowing the estimation of muscle activation with a temporal resolution that would improve the information quality for clinicians in the diagnostic process. In particular, we believe that the proposed method can make an important contribution in the field of rehabilitation allowing to track muscle activation pattern on impaired subject during rehabilitative cycles considering the underlying morphology of the patient and eventual changes that might happen over time due to a pathological situation. The proposed model relies completely on anatomical and physiological information and is therefore easy to understand for clinical personnel, providing results that are giving a direct quantification of the muscle activation. The dimensions of the model are low and this allow a fast estimation process, potentially allowing a real time tracking of the muscle activity, once the model is created from the subject MRI. For this reason, it is a suitable method to acheive a general overview of the neuromuscular system activation status during rehabilitative exercises and therapies, potentially allowing therapists to assess the reaction and the effectiveness of the rehabilitation strategy on the patients.

## Figures and Tables

**Table 1:** Distance error in the projection of the electrodes’ positions. The distance errors in the projection on the selected slice as shown in Figure 4a are reported on first two rows of the table. The distance errors during the projection of the position on the skin surface as shown in Figure 4b are reported on the bottom two rows. For each of the subjects, the mean and the maximum distance errors are reported.

		*Subject 1*	*Subject 2*	*Subject 3*	*Subject 4*
*On the slice*	***Mean distance [mm]***	4.121	3.848	3.167	3.540
	***Max distance [mm]***	11.829	9.029	8.818	7.521
*On the skin*	***Mean distance [mm]***	0.890	0.862	0.789	0.620
	***Max distance [mm]***	2.217	1.980	3.085	1.570

**Table 2:** Goodness of Fit (GOF) value of the reconstructed sEMG value on the electrode space in terms of Normalized Root Mean Square Error (NRMSE).

	Subject 01	Subject 02	Subject 03	Subject 04
middle fing. ext (25% MVC)	96.64 ± 0.41%	95.95 ± 0.39%	96.22 ± 1.27%	97.90 ± 0.17%
middle fing. ext (50% MVC)	97.06 ± 0.55%	95.81 ± 0.34%	98.18 ± 0.26%	98.01 ± 0.09%
ulnar deviation (25% MVC)	94.18 ± 0.57%	94.32 ± 1.60%	90.51 ± 0.43%	95.45 ± 0.53%
ulnar deviation (50% MVC)	96.27 ± 1.34%	95.45 ± 0.84%	89.63 ± 0.67%	96.80 ± 0.53%
wrist ext (25% MVC)	88.94 ± 0.73%	96.43 ± 0.74%	94.55 ± 1.42%	96.27 ± 0.23%
wrist ext (50% MVC)	92.48 ± 0.67%	96.37 ± 0.60%	95.11 ± 1.73%	97.00 ± 0.40%
wrist flex (25% MVC)	93.05 ± 1.67%	95.45 ± 0.44%	91.25 ± 0.45%	90.91 ± 0.90%
wrist flex (50% MVC)	92.91 ± 1.08%	94.78 ± 0.50%	89.25 ± 0.96%	91.46 ± 0.72%

## References

[1] Cagnie B., Elliott J., O’Leary S., D’Hooge R., Dickx N., Danneels L. (2011). Muscle functional MRI as an imaging tool to evaluate muscle activity. J. Orthop. Sport. Phys. Ther..

[2] Franettovich Smith M., Collins N., Vicenzino B. (2019). Intrinsic foot muscle atrophy in individuals with chronic plantar heel pain: a cross-sectional investigation using ultrasound imaging. J. Sci. Med. Sport.

[3] Goubert D., De Pauw R., Meeus M., Willems T., Cagnie B., Schouppe S., Van Oosterwijck J., Dhondt E., Danneels L. (2017). Lumbar muscle structure and function in chronic versus recurrent low back pain: A cross-sectional study. Spine J..

[4] Mayer J.M., Graves J.E., Clark B.C., Formikell M., Ploutz-Snyder L.L. (2005). The use of magnetic resonance imaging to evaluate lumbar muscle activity during trunk extension exercise at varying intensities. Spine.

[5] Dickx N., D’Hooge R., Cagnie B., Deschepper E., Verstraete K., Danneels L. (2010). Magnetic resonance imaging and electromyography to measure lumbar back muscle activity. Spine.

[6] De Ridder E.M., Van Oosterwijck J.O., Vleeming A., Vanderstraeten G.G., Danneels L.A. (2015). Muscle functional MRI analysis of trunk muscle recruitment during extension exercises in asymptomatic individuals. Scand. J. Med. Sci. Sport..

[7] Fernandez-Gonzalo R., Tesch P.A., Linnehan R.M., Kreider R.B., Di Salvo V., Suarez-Arrones L., Alomar X., Mendez-Villanueva A., Rodas G. (2016). Individual Muscle use in Hamstring Exercises by Soccer Players Assessed using Functional MRI. Int. J. Sports Med..

[8] Kinugasa R., Akima H. (2005). Neuromuscular activation of triceps surae using muscle functional MRI and EMG. Med. Sci. Sports Exercise.

[9] Adams G.R., Duvoisin M.R., Dudley G.A. (1992). Magnetic resonance imaging and electromyography as indexes of muscle function. Eur. J. Appl. Physiol..

[10] Drew M.K., Trease L., Caneiro J.P., Hooper I., Ooi C.C., Counsel P., Connell D.A., Rice A.A., Knight E., Hoy G. (2016). Normative MRI, ultrasound and muscle functional MRI findings in the forearms of asymptomatic elite rowers. J. Sci. Med. Sport.

[11] Castelein B., Cools A., Parlevliet T., Cagnie B. (2017). The influence of induced shoulder muscle pain on rotator cuff and scapulothoracic muscle activity during elevation of the arm. J. Shoulder Elb. Surg..

[12] Kumagai M., Shiba N., Higuchi F., Nishimura H., Inoue A. (1997). Functional evaluation of hip abductor muscles with use of magnetic resonance imaging. J. Orthop. Res..

[13] Price T.B., Kamen G., Damon B.M., Knight C.A., Applegate B., Gore J.C., Eward K., Signorile J.F. (2003). Comparison of MRI with EMG to study muscle activity associated with dynamic plantar flexion. Magn. Reson. Imaging.

[14] Merletti R., Botter A., Troiano A., Merlo E., Minetto M.A. (2009). Technology and instrumentation for detection and conditioning of the surface electromyographic signal: State of the art. Clin. Biomech..

[15] Farina D., Holobar A., Merletti R., Enoka R.M. (2010). Decoding the neural drive to muscles from the surface electromyogram. Clin. Neurophysiol..

[16] Holobar A., Minetto M.A., Farina D. (2014). Accurate identification of motor unit discharge patterns from high-density surface EMG and validation with a novel signal-based performance metric. J. Neural Eng..

[17] Holobar A., Farina D., Gazzoni M., Merletti R., Zazula D. (2009). Estimating motor unit discharge patterns from high-density surface electromyogram. Clin. Neurophysiol..

[18] Nakajima Y., Keeratihattayakorn S., Yoshinari S., Tadano S. (2014). An EMG-CT method using multiple surface electrodes in the forearm. J. Electromyogra. Kines..

[19] Su B., Shirafuji S., Oya T., Ogata Y., Funato T., Yoshimura N., Pion-Tonachini L., Makeig S., Seki K., Ota J. Source separation and localization of individual superficial forearm extensor muscles using high-density surface electromyography. Proceedings of the 2016 International Symposium on Micro-NanoMechatronics and Human Science (MHS).

[20] Piovanelli E., Piovesan D., Shirafuji S., Ota J. A Simple Method to Estimate Muscle Currents from HD-sEMG and MRI using Electrical Network and Graph Theory. Proceedings of the 2019 41st Annual International Conference of the IEEE Engineering in Medicine and Biology Society (EMBC).

[21] Piovanelli E., Piovesan D., Shirafuji S., Ota J. Estimating Deep Muscles Activation from High Density Surface EMG using Graph Theory. Proceedings of the 2019 IEEE 16th International Conference on Rehabilitation Robotics (ICORR).

[22] Schneider C.A., Rasband W.S., Eliceri K.W. (2012). NIH Image to ImageJ: 25 years of Image Analysis. Nat. Methods.

[23] Kikinis R., Pieper S., Vosburgh K.G., Jolesz F.A. (2014). 3D Slicer: A platform for subject-specific image analysis, visualization, and clinical support. Intraoperative Imaging Image-Guided Therapy.

[24] Horn B.K.P. (1987). Closed-form solution of absolute orientation using unit quaternions. J. Opt. Soc. Am. A.

[25] Lowery M.M., Stoykov N.S., Dewald J.P.A., Kuiken T.A. (2004). Volume Conduction in an Anatomically Based Surface EMG Model. IEEE Trans. Biomed. Eng..

[26] Dorfler F., Simpson-Porco J.W., Bullo F. (2018). Electrical Networks and Algebraic Graph Theory: Models, Properties, and Applications. Proc. IEEE.

[27] Kapandji A.I. (2019). The Physiology of the Joints.

[28] Platzer W. (2015). Color Atlas of Human Anatomy, Vol.1 Locomotor System.

[29] Kristiansen M., Madeleine P., Hansen E.A., Samani A. (2015). Inter-subject variability of muscle synergies during bench press in power lifters and untrained individuals. Scand. J. Med. Sci. Sport..

[30] Hug F., Bendahan D., Le Fur Y., Cozzone P.J., Grélot L. (2004). Heterogeneity of muscle recruitment pattern during pedaling in professional road cyclists: A magnetic resonance imaging and electromyography study. Eur. J. Appl. Physiol..

[31] Araujo R.C., Duarte M., Amadio A.C. (2000). On the inter- and intra-subject variability of the electromyographic signal in isometric contractions. Electromyogr. Clin. Neurophysiol..

[32] Edwards R.G., Lippold O.C. (1956). The Relation Between Force and Electrical Activity in Fatigued Muscle. J. Physiol..

[33] Naeije M., Zorn H. (1982). Relation between EMG power spectrum shifts and muscle fibre action potential conduction velocity changes during local muscular fatigue in man. Eur. J. Appl. Physiol. Occup. Physiol..

[34] Moritani T., Nagata A., Muro M. (1982). Electromyographic manifestations of muscular fatigue. Med. Sci. Sports Exerc..

[35] Gandevia S.C. (2001). Spinal and supraspinal factors in human muscle fatigue. Physiol. Rev..

[36] Colby Mangum L., Henderson K., Murray K.P., Saliba S.A. (2018). Ultrasound assessment of the transverse abdominis during functional movement. J. Ultrasound Med..

[37] Fukunaga T., Miyatani M., Tachi M., Kouzaki M., Kawakami Y., Kanehisa H. (2001). Muscle volume is a major determinant of joint torque in humans. Acta Physiol. Scand..

